# Bee pollen as a food and feed supplement and a therapeutic remedy: recent trends in nanotechnology

**DOI:** 10.3389/fnut.2024.1371672

**Published:** 2024-06-04

**Authors:** Syed Ishtiaq Anjum, Amjad Ullah, Faryal Gohar, Ghulam Raza, Muhammad Ilyas Khan, Mehwish Hameed, Abid Ali, Chien-Chin Chen, Ivana Tlak Gajger

**Affiliations:** ^1^Department of Zoology, Kohat University of Science and Technology, Kohat, Khyber Pakhtunkhwa, Pakistan; ^2^Department of Plant Protection, Ministry of National Food Security and Research, Karachi, Pakistan; ^3^Department of Biological Sciences, University of Baltistan, Skardu, Pakistan; ^4^Department of Zoology, Abdul Wali Khan University, Mardan, Khyber Pakhtunkhwa, Pakistan; ^5^Department of Pathology, Ditmanson Medical Foundation Chia-Yi Christian Hospital, Chiayi, Taiwan; ^6^Department of Cosmetic Science, Chia Nan University of Pharmacy and Science, Tainan, Taiwan; ^7^Ph.D. Program in Translational Medicine, Rong Hsing Research Center for Translational Medicine, National Chung Hsing University, Taichung, Taiwan; ^8^Department of Biotechnology and Bioindustry Sciences, College of Bioscience and Biotechnology, National Cheng Kung University, Tainan, Taiwan; ^9^Biotechnology Center, National Chung Hsing University, Taichung, Taiwan; ^10^Department for Biology and Pathology of Fish and Bees, Faculty of Veterinary Medicine, University of Zagreb, Zagreb, Croatia

**Keywords:** bee pollen, composition, bee pollen vitality, apitherapeutic, bee pollen consumption and nanotechnology

## Abstract

Pollen grains are the male reproductive part of the flowering plants. It is collected by forager honey bees and mixed with their salivary secretions, enzymes, and nectar, which form fermented pollen or “bee bread” which is stored in cells of wax honeycombs. Bee pollen (BP) is a valuable apitherapeutic product and is considered a nutritional healthy food appreciated by natural medicine from ancient times. Recently, BP has been considered a beneficial food supplement and a value-added product that contains approximately 250 different bioactive components. It contains numerous beneficial elements such as Mg, Ca, Mn, K, and phenolic compounds. BP possesses strong antioxidant, anti-inflammatory, antimicrobial, antiviral, analgesic, immunostimulant, neuroprotective, anti-cancer, and hepatoprotective properties. It is used for different purposes for the welfare of mankind. Additionally, there is a growing interest in honey bee products harvesting and utilizing for many purposes as a natural remedy and nutritive function. In this review, the impacts of BP on different organisms in different ways by highlighting its apitherapeutic efficacy are described.

## 1 Introduction

Bee pollen (BP) is a raw flower plant reproductive material. In the 17th century, the term pollen was established from a Latin word which means fine powder or flour. For centuries, this plant component has been known as a “food” with biological functions (1). Honey bees rely on pollen for proteins, lipids, fatty acids, sterols, carbohydrates, and vitamins (2, 3). Honey bee foragers shape field-gathered pollen in pellets and transport it to a hive that produces a high-quality product called bee bread (4–6). Pollen stored in honey bee wax combs after lactic fermentation in a hive is referred to as bee bread or fermented pollen (7, 8). It is obtained as a result of lactic acid fermentation of pollens by some bacteria and fungi (4–6). In addition, corbiculate bees add regurgitated fluid with the grains to facilitate pollen adherence to the pollen baskets and pollen grains (8–10). BP is collected by adult forager bees and transported to the hive. The flightless house bees fragment the mixture of collected pollen, saliva, honey, digestive enzymes, and lactic acid, which is then stored in wax honeycomb cells. After collecting pollen, it is fermented, stored, and covered with a thin layer of wax and honey (11). The bee bread undergoes anaerobic lactic fermentation during the ripening process, which is caused by bacteria (e.g., *Lactobacillus* spp., *Pseudomonas* spp.) and yeasts (*Saccharomyces* spp.). Bee bread is the primary source of protein for honey bee colonies and also provides minerals, vitamins, and other nutrients needed for the production of royal jelly (11–13).

Approximately 50 to 250 grams of pollen can be collected by forager bees of one colony per day, while 14 to 40 kilograms of pollen collected by forager bees each year (4, 14). BP preservation can be achieved by drying for a longer period at room temperature in special driers. This helps to prevent microbial spoilage and rapid fermentation, resulting in easy marketability and increased revenue for beekeepers (2, 15). Furthermore, BP with high moisture content easily spoils in a short period after harvesting because it is highly vulnerable to microbial attacks (2, 16). Two pollen pellets have a combined total mass near 20 mg (17), which is more than 25% of the average body mass of the honey bee (17, 18). Bees that collect pollen do not consume pollen from the flower but they take it for the consumption of their offspring in the hive and behave as social bees (9).

Honey bee products are highly recommended and used worldwide for various purposes, such as natural food, medicine, and cosmetics, and their importance was revealed in the Holy books (19). BP is a nutritional and functional bee product that is used as an apitherapeutic product and food supplement. The composition of BP depends on flower source, season, geographical origin, and plant species. BP contains carbohydrates, proteins, lipids, phenolics, vitamins, and minerals in its composition. BP has many pharmacological properties and can be used to treat metabolic abnormalities such as hyper-dyslipidemia, obesity, diabetes, and cardiovascular diseases (20, 21). BP can be found in the form of pills, granules, oral liquids, tonics, and candy bars (22). Moreover, BP can be used as a food supplement due to its antioxidant, hepatoprotective, antimicrobial, cardioprotective, and immunomodulatory properties (23, 24). The main aim of the study was to highlight the benefits of BP regarding health and its efficacy toward different human and animal health problems.

## 2 Composition and morphology of bee pollen

The BP composition generally depends on plant species, geographical origin, and seasonal conditions. Generally, BP consists of carbohydrates (40–85%), proteins (14–30%), lipids (1–10%), vitamins, minerals, carotenoids, phenolics, and other trace elements (14, 20, 25, 26) (Table 1). A total of 250 bioactive compounds in BP have been known so far (44–47). The characterization of the BP according to the parameters of antioxidant activity, botanical origin, and phenolic profile was previously described (48). The chemical composition of BP is known to vary with the used methods of extraction and storage, the floral origin, and environmental conditions (5, 49, 50). Pollen comes in different colors depending on botanical origins, such as green, black, grey, purple, orange, white, and red (44, 51, 52). Pollen grains collected of the same color are said to be monochromatic while having different colors called polychromatic BP (53). Colored pollen (anthers) might act as an additional visual indication, helping the foraging insect pollinators to detect the flower over a small distance (52, 54). BP collected from different flowers is called multifloral while collection that takes place from a single flower or plant is said to be monofloral (55).

**Table 1:** Chemical composition of honey bee pollen.

Components	Sub-components	References
Carbohydrates	Sucrose, Glucose, Fructose, Maltose, Melezitose, Isomaltose, Trehalose, Taffinose, and Erlose	(14, 25, 27–29)
Protein	Leucine, Isoleucine, Aspartic acid, Threonine, Proline, Valine, Phenylalanine, Lysine, Tryptophan, Asparagine, Serine, Glutamine, Glutamic acid, Glycine, Cystine, Tyrosine, Histidine, Alanine, and Arginine	(25, 30–32)
Lipids	Caproic acid, Margaric acid, Caprylic acid, Capric acid, Oleic acid, Lauric acid, Myristic acid, Pentadecylic acid, Palmitic acid, Erucic acid, Gadoleic acid, palmitoleic acid, Stearic acid, Linoleic acid, α-Linolenic acid, Behenic acid, Arachidic acid, Eicosatrienoic acid, Nervonic acid, and Lignoceric acid	(25, 31, 33, 34)
Vitamins	Vitamin A (β-Carotene), Vitamin B1, B2, B3 (Nicotinic acid, Nicotinamide, Niacin), Vitamin B5, B6 (Pyridoxol, Pyridoxal, Pyridoxal, Pyridoxine), Vitamin B9 (Folic acid), Vitamin B12, Vitamin C (Ascorbic acid), and Vitamin E (α-Tocopherol)	(25, 27, 31, 35)
Minerals	Potassium, Copper, Sodium, Calcium, Magnesium, Phosphorus, Manganese, Strontium, Zinc, Copper, Aluminum, Manganese, and Iron	(25, 30, 31, 36, 37)
Phenolics	Gallic acid, Epicatechin, Protocatechuic acid, Catechin, Quercetin, Naringenin, Kaempferol, Luteolin, Hesperetin, *tert*-Cinnamic acid, *o*-Coumaric acid, Ferulic acid, Benzoic acid, *p*-OH benzoic acid, Chlorogenic acid, Rutin, Caffeic acid, *p*-Coumaric acid, Vanillic acid, Syringic acid, 2-Hydroxycinnamic acid, Apigenin, 3,4-Dimethoxycinnamic acid, Quercetin-3-O-*β*-D-glucosyl-(2 → l)- *β*-glucoside, Kaempferol-3,4′-di-O-*β*-D-glucoside, Kaempferol-3-O-*β*-D-glucosyl-(2 → l)-*β*-D-glucoside, Isorhamnetin, Quercitrin, Quercetin 3-O-glucoside, and Kaempferol 3 O-glucoside	(25, 38, 39)
Carotenoids	Lutein, *β*-cryptoxanthin, *β*-carotene	(4, 25, 33, 40, 41)
	α-Carotene, γ – Carotene, ξ – Carotene, *ε* – Carotene, Lycopene, Isocryptoxanthin, Zeaxanthin, Capsantine, Neoxanthin, Canthaxanthin, Astaxanthin, Isozeaxanthin, Lactucaxanthin Lutein, Antheraxanthin, and Violaxanthin	(4, 41–43)

In closely related plant species, the amino acid composition of their pollen is similar, while in distantly related plant species, the amino acid composition differs (56, 57). Pollen mixing may dilute the potentially toxic compounds originating from toxic plants (58). In the BP, the predominant minerals are calcium, magnesium, manganese, and potassium. The BP is considered a rich source of various vitamins, including B1, B2, and B6 (3, 59). The composition and quantity of amino acids in pollen proteins depend on the plant species visited by honey bees (60).

## 3 Factors affecting bee pollen

### 3.1 Plant species effect

The dietary component of pollen grains differs strongly among various plant species and is directly related to honey bee health (61). Honey bees and bumble bees detect and make decisions about foraging based on the nutritional content of floral resources (62). Bees often grasp flowers with their antennae while sometimes grasping and scraping pollen from the anthers with the help of their mandibles (63). Even some species have mouthparts with specialized hairs intended for pollen collection from flowers (63, 64). If honey bees collect pollen from a single plant species, it is called monofloral (at least 80%) (45, 65, 66); if they collect from multiple species, it is called multifloral (less than 80%) (4, 53, 65, 66). According to the research, honey bees have a preference to collect pollen from 2 to 8 plants every single month (67). BP with small amount of protein content can have adverse effects on health of bees, such as reducing hypopharyngeal gland size in adult honey bees (68, 69), low larval weight in bumblebees (*Bombus terrestris*) (69, 70), sweat bees (*Lasioglossum zephyrum*) offspring weight (69, 71), and immune function in honey bee colonies (69, 72, 73).

### 3.2 Geographical effects

Climatic conditions and the pollen ripening period influenced the quality of pollen and the vitality of pollen during the years of cryopreservation. On the vitality of pollen cryopreservation differences between the genetic variability of trees (inter-individual variability) were very pronounced and enabled the selection of trees that are to be pollinated by insects and which keep their initial vitality (74). The composition of pollen may vary in different annual seasons, years, and different geographical locations (49). BP has an extensive chemical nutritive composition but the composition has variation in the maximum and minimum range of values, especially because of different botanical origins, edaphoclimatic, and geographical conditions (75). However, because of geographic and botanical origin and other factors such as beekeepers’ practices, weather conditions, and soil type, there is a variety in BP composition (76).

### 3.3 Temperature effects

The viability of pollen can decrease by 30–70% under heat stress. Pollen wall thickness increases under heat stress (77). *In vitro* germination was used to assess the viability of pollen and it was measured at intervals up to 2 h with the removal of pollen grains from the anthers. It was quantified for various alfalfa plant (*Medicago sativa*) varieties at three temperatures including both, Roundup Ready (RR) and conventional varieties. The most prevalent factor affecting the viability of pollen in alfalfa was the time since the removal from the anthers. The viability of pollen declines with increasing time for all tested varieties at all three temperatures. Additionally, the viability of pollen is not affected by the temperatures ranging between 25 and 37°C and does not vary among varieties of plants, including RR and conventional varieties (78).

## 4 Apitherapy

**Figure 1:**
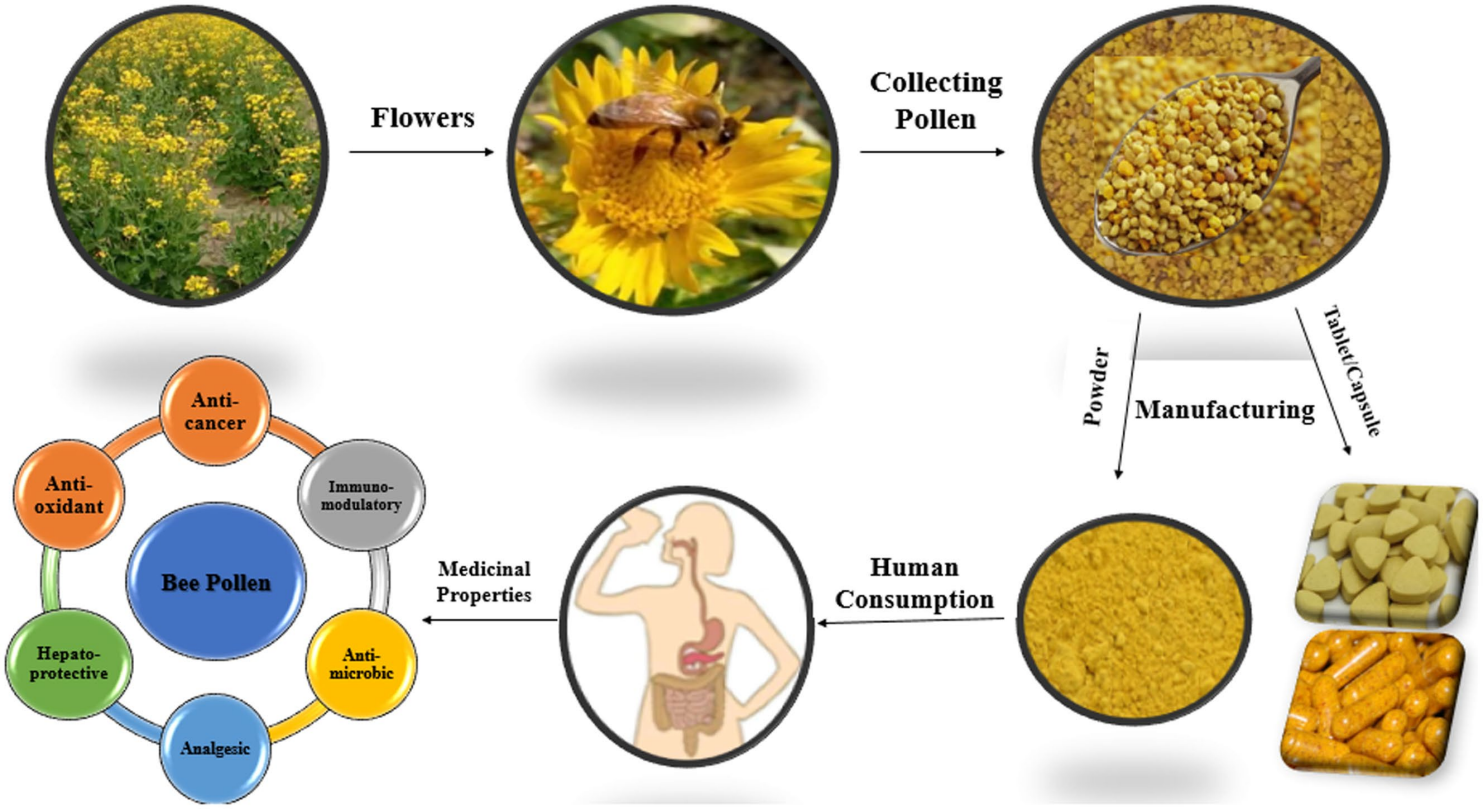
Bee pollen collection/composition and medicinal properties.

**Figure 2:**
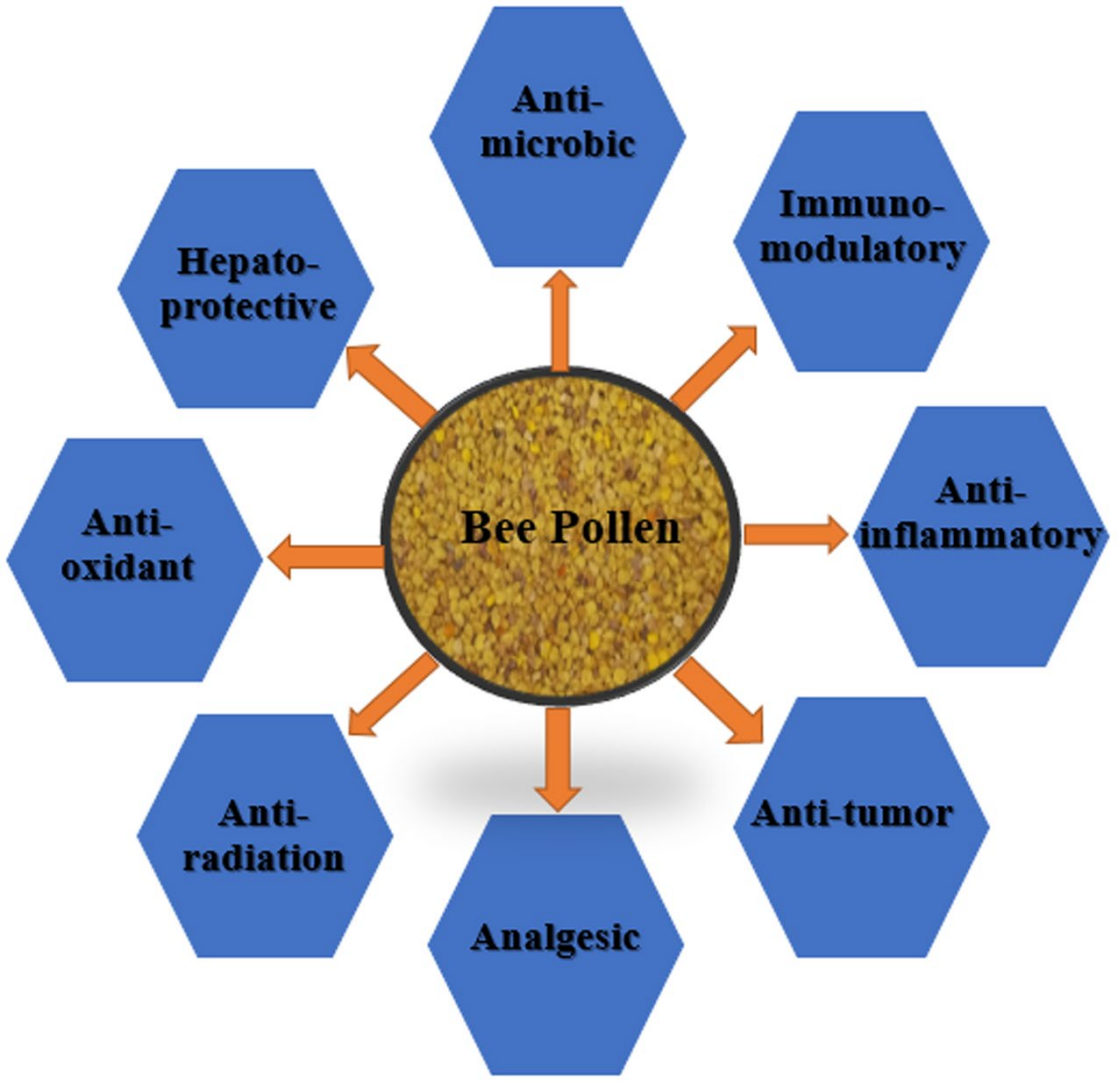
Schematic illustration of bee pollen therapeutic properties.

A zootherapy type called “apitherapy” is based on the bioactivity of several chemical compounds from honey bee products (79, 80). Apitherapy contains a number of chemical compounds which are natural agents with an approved range of activities and actions (11, 81). BP, a commonly used apitherapeutic, varies in chemical composition depending on geographic origin, plant source, soil type, bee race, activities, and climatic conditions (8, 11, 30, 46, 81). BP shows abundant therapeutic and nutritional properties because of the richness of its bioactive and nutritive components (82). Pharmacopoeia Committee of the People’s Republic of China officially recognized BP as a healthy food. It has been concluded that BP can delay aging, improve the cardiovascular system, maintain the digestive system, prevent prostate degeneration, and enhance immunity according to a previously published article (76, 83). In the apitherapeutic treatment, BP is used because it shows medicinal activities such as antimicrobial, antibiotic, anti-inflammatory, antiviral, analgesic, immunostimulant (84–86), anti-radiation, anti-tyrosinase, hepatoprotective, and antioxidant (22, 87–89) (Figures 1, 2).

Pollen consumption can lower blood pressure and increase hemoglobin and red blood cells. It may benefit those with sterility, hypertension, and pernicious anemia, as well as the endocrine system and nervous system (90–92). BP is rich in protein, fat, and minerals (particularly Ca, Mg, Fe, and P) and gives a nutritional value to pollen similar to or greater than dry legumes and is used as a food supplement (93, 94). In other studies, restorative, positive, and protective effects of pollen addition in feed have been reported (95). The pollen proteins improve regeneration of liver tissue and increase levels of serum albumin and state of malnutrition in cirrhotic rats when compared with the untreated group (95, 96). In diabetic rat, bee bread beneficially affects the intake of water and metabolism of glucose and is helpful for the cure of other diseases in diabetes and hyperglycemia (97). It has been studied that the use of BP extract considerably reduces the level of phosphate and calcium in urine which avoids kidney stone formation in fed rats (98).

### 4.1 Antioxidant activity of bee pollen

BP contains antioxidants with low molecular weight compounds. Among them, the most important compounds are ascorbic acid (vitamin C) and phenolics which are associated with the hydrophobic class of antioxidants (99, 100), whereas carotenoids and tocopherol (vitamin E) belong to hydrophobic antioxidants. Additionally, vitamin C reacts with hydroxyl radicals as it is a water-phase antioxidant. It helps in retaining the NO (nitric oxide) level in oxidative stress, and the compound can help in the relaxation of smooth muscles of arteries (100–102). Additionally, it was found that the fraction of ethyl acetate of camellia (flowering plants) BP possesses higher anti-tyrosinase and antioxidant potential than other pollen such as rose BP, lotus BP, and rape BP (87). It has been noted that the fermentation of BP can increase its phenolic compound contents and promote its antioxidant capability (21). Furthermore, BP was added to beef burgers and examined its oxidative stability, chemical composition, and antioxidant properties. Using TBARS (thiobarbituric acid reactive substances) assay method, it was noticed that the malondialdehyde value per kg of beef burger enhanced up to 2.09 mg at −12°C after 42 days of storage. In addition, BP extract repressed 31.78% of lipid oxidation of beef burgers at the end of the analyses (59). Therefore, due to its strong antioxidant activity and high nutritive value, BP may be used as a natural anti-oxidant in meat products (59, 103). Similarly, lyophilized BP also has strong antioxidant capability in pork sausage (103). Another study concluded that the addition of BP to broiler meat can increase water decrease fat contents and ameliorate meat quality which is functional for human health (104). Anjos et al. noted that BP inhibited lipid oxidation in black pudding and enhanced product quality in Portugal (48, 59). The addition of BP to meat products can increase their preservation time due to the presence of quercetin and other derivatives in BP which acts as an antimicrobial agent. Furthermore, the usage of BP (high amount) in frankfurters can increase its oxidative stability and shelf life during chilled storage which is attractive to potential market consumers (105). It has been found that BP decreases the toxic effect of fluorine (due to its antioxidant activity) via the reduction of MDA levels in albino male rats. In addition, it also enhanced the total protein level, phosphorous, calcium, magnesium, and serum level than the fluoride-treated control group. The mixing of 0.5 to 3% BP (ground form) with yogurt (made from sheep, goat, or cow milk) significantly increases its antioxidant capability and phenolic compounds and also ameliorates its smell, taste, and appearance (20). Furthermore, the addition of BP to malt beverages significantly enhances the antioxidant action because of the presence of a greater quantity of phenolic contents, especially *Papaver somniferum* (poppy) pollen than the control group (106).

The ratio of polysaturated fatty acid in pollen is higher than the saturated fatty acid and monosaturated fatty acid (107, 108), mainly depending on plant species and storage conditions (109, 110). Dietary supplementation with BP expresses a positive effect on stress, reducing lipid peroxidation due to the presence of antioxidants in its composition (107, 108) and increasing polyunsaturated fatty acid in some tissues due to higher polyunsaturated fatty acid content. The polyunsaturated fatty acid dietary supplements have a beneficial impact on the antioxidant status of consumers (108, 111) and change in the health and performance of Japanese quails (*Coturnix coturnix japonica*) (108). It has been stated that the gastrointestinal infection of broiler chickens was cured through BP (45). The addition of 1.5% BP to the feed of broiler chicken significantly increased immunoglobulin M (IgM) concentration and weight of thymus after 21 and 42 days, respectively, and enhanced the immune system of chicken (at 21 days of age). In another study, it was shown that the presence of antioxidants such as luteolin, apigenin, and quercetin in BP considerably improved intellectual activities and functions (20).

### 4.2 Anti-inflammatory activity of bee pollen

The components of BP include proteins, minerals, lipids, vitamins, carbohydrates, and some other components in a small amount. Previous studies have revealed that BP has antibacterial, antioxidant, anti-carcinogenic, anti-allergic, and anti-inflammatory properties (25, 36, 46). Both BP and propolis are rich in trace elements, flavonoids, and other healthy components and have been shown to have many health benefits, such as anti-allergic and anti-inflammatory properties (112). In addition, BP is also rich in quercetin, which has been found to affect the metabolism of arachidonic acid and decrease the level of pro-inflammatory prostaglandins (22, 113). Due to its anti-inflammatory properties, quercetin also targets cyclooxygenase 1 and cyclooxygenase 2, following the obstruction of NFκB processes (114). Related to inflammation and oxidative stress, phenolic compounds present in many medicinal plants play an important role in treatment. Therefore, antioxidant activity may be one of the possible mechanisms of the anti-inflammatory effect of BP (115). Moreover, BP suppresses and downregulates the expression of NF-κB (induced nuclear factor) and tumor necrosis factor (TNF) due to its anti-inflammatory activity (84). Yeast-fermented BP possesses hepatoprotective efficacy via reducing malondialdehyde (MDA) levels while boosting the amount of catalase (CAT) and glutathione *S*-transferase (GST) in the mice liver (116). Both BP and bee bread possess a flavonoid called isorhamnetin which has anti-cancer and anti-inflammatory properties. Kaempferol is another phenolic compound which is known for its therapeutic potential and used for the additional therapy of hepatic fibrosis (4).

Furthermore, the component of BP called rutin has been used in preparation that acts as anti-inflammatory, neuroprotective, and antispasmodic agents (89). The presence of glucoside and flavone in BP makes it a nutraceutical agent which has therapeutic efficacy against various diseases such as amnesia, Alzheimer’s disease, and diabetes (117). Treatment with BP significantly diminished inflammation and TNF-α levels in polycystic ovary syndrome in mice model (118). Tyrosol is another BP (*Rhododendron ponticum*) phenolic component that possesses anti-cholinesterase and anti-hypoglycemic function and also restrains Parkinson’s and Alzheimer’s diseases (119). It was investigated that the component of BP called linalool holds anti-inflammatory characteristics that are correlated with the suppression of pro-inflammatory variation of mitogen-activated protein kinases (MAPKs) and NF-kB pathways (6). The BP fatty acids called linoleic and linolenic combine with histamine H1 receptor while flavonoids decrease the production of nitric oxide and both act synergistically by diminishing the inflammatory responses (120). Furthermore, BP significantly suppresses oxidative stress and lipid peroxidation and neuroinflammation in the hippocampus tissues of mice brain (121). Moreover, the chemical composition of BP and other bee products is complicated and diverse and depends on many biological factors. While its clinical applications are still limited, ongoing future research will validate and consider BP hopefully as a clinical therapeutic remedy with promising healthy outcomes.

### 4.3 Anti-microbial activity of bee pollen

The extracts of both BP and bee bread possess anti-bacterial activity toward gram-positive bacteria and gram-negative bacteria. However, the extract of bee bread (minimum inhibitory concentration of 2.5 to 10% v/v) has higher antibiotic potential against *Staphylococci* species than bee pollen extract (minimum inhibitory concentration of 5% to >20% v/v). It was further studied that gram-positive bacteria were more susceptible to both extracts as compared with gram-negative bacteria (122). BP ethanol extracts show quite strong antibiotic properties against pathogenic fungi, gram-negative bacteria, and gram-positive bacteria because it contains phenolic acids. Flavonoid effects on bacteria are related to their metabolic disruption. The mechanism involves the formation of complexes with the cell wall of bacteria by surface-exposed polypeptides or cell membrane enzymes and adhesion, which blocks ion channels and inhibits electron flow in the electron transport chain which determines adenosine triphosphates (ATP) production by electron scavenging and also the disruption of cell wall integrity (100). By the use of a suitable extraction solvent, the bioactive properties of the honey BP can be increased (123). Additionally, both BP and bee bread contain beneficial bacterial and fungal species that have antimicrobial potential against some human clinical bacterial isolates such as *E. coli*, *Staphylococci* spp., and *P. aeruginosa* (124). Ethanol and methanol extracts of BP have similar antibacterial action against described bacteria. In addition, they show antifungal activity against *Aspergillus flavus*, *A niger*, and *A fumigatus*, and yeasts such as *Rhodotorula mucilaginous*, *Candida krusi*, *C glabrata*, and *C albicans* (100, 125). It has been stated that the number of *Lactococcus* species raised in the oral microbe’s community in the mice model (BP group) has antimicrobial activity against *P. gingivalis* and *S. mutans* (biofilm-forming bacteria) species. Thus, BP can protect and have an expedient impact on the oral cavity and the gastrointestinal tract (84). Recent studies reported that the BP from Wadi Al-Nahil showed antibacterial activity (*in vitro*) toward *Clostridium perfringens* (a pathogenic bacteria present in autism patients higher than in healthy individuals) (36).

It was examined that BP collected from *Attalea funifera* can have leishmanicidal activity against amastigote and promastigote types of *Leishmania amazonensis* due to various active compounds (126). The addition of BP to meat products can increase its shelf life because of its natural antimicrobial and antioxidant activities (4). The component of BP called rutin has been used in drug that acts as antibacterial, antiviral, and cardio-protective agents (89). In a recent study, five types of Portuguese BP were used against both types of bacteria (gram-positive bacteria and gram-negative bacteria) and yeasts which revealed growth inhibitory and antimicrobial action (50). In contrast, some BP extracts were examined against gram-negative bacteria which exhibited antibiotic action such as *Acinetobacter baumannii* and *E. coli*, while *K. pneumoniae* and *P. aeruginosa* showed resistance (127). It was also noted that the BP from *Cistus* and *Castanea* plants possesses higher antimicrobial activity as compared with *Rubus* (flowering plants), which may correlated with the high content of phenolics such as hydroxycinnamic acid (50). It has been stated that the fermented BP possesses strong antifungal action toward *Penicillium roqueforti* (128). A few studies revealed that the phenolic content of BP has a positive impact on the growth of useful gut microbiota while inhibiting the development of pathogenic microbes (45). Due to BP’s antimicrobial activity, it is also used in toothpaste, which inhibits germ proliferation and reduces oral inflammation (22).

In the current era, an excessive number of antibiotic drugs have been used against various diseases and pathogens which created an alarming situation because several pathogens showed resistance toward these antibiotics. Researchers throughout the world trying to investigate natural products (nutraceuticals) that are non-resistible and environmentally friendly (129–131). Like other beneficial products of animals and plants, bee hive products such as propolis, royal jelly, bee venom, and BP are well recognized as a natural remedy and considered healthy and supplemental food products (132–134).

### 4.4 Impact of bee pollen on skin health

From ancient times, beneficial effects of BP on skin conditions have been known. Sun et al. also studied the effect of free phenolic extract of BP on melanogenesis using B16 mouse melanoma cells (76). The melanin relative content and intracellular tyrosinase activity decreased in a very distinct and dose-dependent way by the studied substance. Free phenolic extract of BP expresses great effectiveness by increasing the reducing power that indirectly contributes to the reduced synthesis of melanin, which shows the usefulness of BP by protecting the cell from abnormal production of melanin (76, 135). Furthermore, Korean scientists also confirmed BP’s usefulness in the manufacturing of cosmetics that protect the skin from oxidative stress and hyperpigmentation (135, 136). BP is used in the form of lipid, aqueous, and lyophilized extracts. Furthermore, active substances can be extracted with oils, glycerin, water, and propylene glycol. BP extracts are used in cosmetics in a concentration of 0.5–5%. The high content of flavonoids has a significant effect on skin tissues which allocates the BP to seal and strengthen the capillaries caused by higher vitamin C content. In addition, BP stimulates mitotic division, enhances cell metabolism, and improves regeneration. Due to the occurrence of phospholipids, zinc, and methionine, BP normalizes the action of sebaceous glands. Moreover, BP strengthens the hair shaft because of sulfur-containing amino acids (137, 138).

BP has conditioning, regenerating, and moisturizing properties, stops the scalp itching and limits the growth of fungus, and is also added to the anti-dandruff shampoo (22, 138). Researchers studied that the best solution would be to mix the BP with ethyl esters of essential unsaturated fatty acids from flaxseeds. Additionally, BP may effectively increase safe mechanisms against skin dryness, oxidative damage caused by ultraviolet radiation, melanogenesis and inflammation have a role in a great variety of negative effects on human skin (139). Studies have shown that 1% BP can decrease lactate dehydrogenase release by 18.73% of dermal fibroblast. A clinical study revealed that BP had useful effects on wrinkles around the eyes, skin roughness, transparency, and hydration. Furthermore, there are no adverse effects on the skin by the BP (140). In addition, BP ointment was applied in the topical burn treatment for the first time (141). On two white domestic pigs, burn wounds were imposed and treated with bee pollen or silver sulfadiazine ointment. The comparative material was constituted by either tissue which was treated from a wound or tissue obtained from wounds treated with physiological saline. Histopathological and clinical evaluation revealed that applied apitherapeutic product decreases the burn wound healing time and positively affects the general condition of an animal. Moreover, the BP ointment may have a positive impact on the process of healing burn wounds, avoiding newly developed tissues from infection (142).

Rape BP phenolic extracts showed strong anti-tyrosinase activity *in vitro* (76). Studies have shown that phenolics are found in free and bound form (esterified and insoluble bound form). Both phenolics are produced in the endoplasmic reticulum and transported to the vacuole (free form) and cell wall matrix (bound or insoluble form) (143, 144) where they attached (via covalent, ester, and ether bonds with a cell wall) with arabinoxylans, pectin, cellulose, structural proteins, and hemicellulose of the plant cell (143, 145). Phenolic compounds have a major role in plant cells, i.e., protecting cells from UV rays of sunlight and insect predators (producing annoying/toxic substances) and penetration of pathogens (fungi) in addition to attracting plant pollinators (143, 146). Free-form phenolic activities were greater than the bound form. Free phenolic extracts of rape BP can be used as a natural anti-melanogenesis composition source (76). Furthermore, the components of BP such as kaempferol, levulinic acid, 5-hydroxyfurfural, phenolamides, sterols, *p*-coumaric acid ethyl ester, and caffeine exhibit anti-tyrosinase activity. However, phenylamides were regarded as a more powerful anti-tyrosinase agent than the other pollen components (147). It has been documented that BP from a corn tree (*Quercus acutissima*) is reported as an excellent antioxidant and anti-melanogenesis agent due to the presence of high amount of phenolic acids, and its possible mode of action is the obstruction of melanin-producing enzyme (tyrosinase) (22).

On the other hand, free phenolics are digestible in the small intestine while bound phenolic compounds are not absorbed (in the small intestine) and reached in the large intestine where they pass the fermentation process (by colon microbiota) and liberate bound phenolic compounds. Additionally, these phenolics have a great role in the colon by inhibiting cancer-causing microorganism growth via reducing pH in the fermentation environment (143, 148). Moreover, free phenolic extract from rape BP possesses higher capabilities to defend body cells from aberrant melanogenesis than bound phenolic extract via the suppression of microphthalmia-associated transcription factor (MITF) and downregulation of cyclic adenosine monophosphate (cyclic adenosine monophosphate) while preventing the anti-tyrosinase and antioxidant pathways *in vitro* (22). The methyl gallate and gallic acid (phenolic acids) obtained from *Givotia rottleriformis* diminish the growth of skin cancer cells (human epidermoid carcinoma) (149) and restrain hepatitis C virus (HCV) which causes hepatocellular carcinoma and liver cirrhosis (143, 150). In addition, a flavonoid called kaempferol possesses many biological activities such as cardio-protective, anti-inflammatory, anti-allergic, antidiabetic, osteoporotic, and analgesic (143, 151). The valuable effects of BP on human skin are well recognized so far. In addition, the negative impact of chemical cosmetics on human skin health warned people to use natural products that have both properties of chemo-protective and healthy dermal maintenance.

### 4.5 Effect of bee pollen on animal immunity

BP comprises over 50% more protein than beef; however, its lipid content is very low. To reduce the radiation effect and retard aging and also improve the immune response, pollen may be used because of its flavonoid and antioxidant components (152). The addition of BP in the diet of freshwater fish has led to improved immune status and growth, reduction of the mortality caused by *Aeromonas hydrophila,* and increase in the number of phagocytic cells (i.e., neutrophils and monocytes) in Nile tilapia (*Oreochromis niloticus*) (153). In the Nile tilapia, the therapeutic mode of action of BP was detected on pathogen *A. hydrophila*, where all treated fish showed significant protection. In detail, the experimental group fed with 2.5% (w/v) BP for 20 and 30 days showed the greatest protection against the described pathogen. In addition, BP increased significantly the growth performance parameters [length, average daily weight (ADG), body weight, feed efficiency ratio (FER), and specific growth rate (SGR)], as well as biochemical (albumin, globulin, and serum total protein ratios), immunological (serum bactericidal activity), nitro blue tetrazolium assay (NBT), and hematological parameters [hematocrit (Hct), leucrit (Lct)] as the number of lymphocytes, neutrophils, monophils, and phagocytic activity (154). Pollen and propolis extracts used for rainbow trout (*Oncorhynchus mykiss*) as a food additive had a positive effect on its final consumption weight and growth performance (155). Moreover, BP polysaccharides from corn enhance the activity of immunocytes in the body such as lymph nodes, spleen, thymus gland, and bone while strengthening immunity of the body against pathogenic microbes (22).

The fish diet added with the mixture of pollen and propolis (0.5%) was more effective with an increase in weight and a decrease in feed consumption as compared with the fish fed with pure pollen or propolis. This growth performance resulted from the mixture of propolis and pollen could be due to their components such as minerals, enzymes, or coenzymes as well as vitamins which enhance food digestion (156, 157). The diet enriched with a mixture of 0.5% propolis or 0.5% pollen is more effective than 1% propolis or 1% pollen alone on hematological parameters, plasma, and growth performance of rainbow trout (157). During the summer season under climatic conditions with very high air temperatures, the BP treatment impacts the hematological, physiological traits, and productive performance of rabbit bucks (158). An obvious improvement in immune status parameters was recorded after the supplementation of BP in diabetic rats as an antioxidant-rich food supplement (159). BP consumption significantly enhances the phagocytic capacity and index in the granulocytes of rabbits (160, 161). Previous studies revealed that the polysaccharides of BP (Chinese wolfberry) have immunomodulatory activity *in vitro* against RAW 264.7 cells via downregulating the level of tumor necrosis factor-alpha (TNF-α), interlukin-1 (IL-1), interlukin-6 (IL-6), and nitric oxide (NO) secretion (162).

Here, the consumption of BP led to the greatest increase in *ex vivo* proliferation of lymphocytes, while these results could simply be related to the presence of a great concentration of vitamins, trace elements, and amino acids that stimulate differentiation and proliferation of immune cells (161, 163). It is also possible that T lymphocyte formation is stimulated by the polysaccharide constituent in the BP (161). Various research studies have been done regarding pollen as a rich source of food for animals. Researchers concluded that pollen is a complete and functional food source because it contains important nutrients such as phenolics, vitamins, minerals, and other metabolites which are immune boosters, and food supplements have a positive impact on physiological health and possess promising therapeutic capabilities.

### 4.6 Effect of bee pollen on the animal reproductive system

The BP supplementation with 0.2 g per kg of body weight in the rabbit diet improved fertility (86.9%) of the control group (69.5%). By using BP mixed with propolis (100 + 100 mg/kg body weight), the experimental group showed increased body weight, the number of live kits (baby rabbits) at birth (*p* < 0.01) than the other groups, and greater litter size, but the number of stillborn kits did not differ significantly among all groups (164, 165). The complementary result of the supplementation of BP diet for rabbits on productive traits might be accredited to the high amount of macronutrients and micronutrients, e.g., minerals, in addition to phytosterols (165, 166). The enhancement of reproductive properties in rabbits might be related to the ability of BP to enhance estrogen levels appropriately and balance the hormones needed for conceiving. In addition, BP boosts the ability of eggs (in female rabbits) to withstand and survive during the period of incubation (165). The antigenotoxicity and genotoxicity as well as antiestrogenic and estrogenic activity of *Cystus incanus* L. and *Salix alba* L. of BP and its processed extracts in human and yeast cells were tested, and it was found that they were neither estrogenic nor genotoxic and able to reduce the damage of chromosome induced by the used cancer drugs, thus supporting their use as a chemoprotective agent (95, 96). BP and mannan oligosaccharides improved the antioxidant status and semen quality of rabbit bucks during the Egyptian summer season (167). Studies have shown that BP individually or coupled with metformin effectively suppressed the levels of TNF-α, Ki67, and NO while boosting mature follicles and P53 in polycystic ovary syndrome-treated rats (118).

Rabbit bucks raised under high temperature and treated with 1,000 mg and 500 mg of BP per buck, significantly ameliorating blood parameters, testosterone hormone, antioxidant activity, and semen characteristics (168). Ensuring animal welfare, increasing productivity, and improving product quality are common demands of animal breeders. Pollen and propolis can be added to the diet of laying quail to improve egg quality and enhance blood protein, egg production, lipid, and immunological responses (169). In the diet of Sinai chicken, BP could be used at 1000 mg/kg to ameliorate the live sperms, while the sperm aberrations were considerably decreased to approximately 36.28% than the control group (170). The BP (collected from date palm) was given orally to male Wister rats (having induced diabetes via streptozotocin) with a dosage of 100 mg/kg (daily up to 4 weeks), and considerable enhancements were detected in malondialdehyde (MDA), testicular nitric oxide, and blood glucose levels. As a result, significant development was observed in follicle-stimulating hormone (FSH), luteinizing hormone (LH), pancreas weight, serum insulin levels, body weight, testosterone, sperm viability, and motility than the diabetic control group. The administration of BP regulates the apoptotic function and secretion from ovaries of Wistar rats (40-days-old females who are clinically normal) (20, 22, 171).

It has been documented that the dose of 60 mg/day per animal of BP to a mice model (Turkish origin) significantly enhanced the sperm counts and testosterone levels for 1 month. Similarly, the treatment of lead-induced mice model with BP (Algerian origin) (100 mg/kg) noticeably improves the spermatogenesis process while demolishing sertoli cells (171). The presence of gonadotropin hormone in the date palm pollen surprisingly raised the possibility of the treatment of sterility. Many animal studies revealed that BP enhances semen quality and quantity as well as improves physiological health and other chemical substances that take part in the reproduction process of an individual organism. In addition, the human population also has some fertility and sexual problems, which can be treated using BP as a natural reproductive medicine and innovative food supplement.

### 4.7 Anticancer properties of bee pollen

Cancer is one of the known diseases worldwide and a major cause of human disease. It has been stated that the cytotoxicity effect of BP is mainly linked with flavonoids and phenolic compounds present in its chemical composition. The mechanism of anti-carcinogenic activity of these components is due to the secretion of TNF-α and stimulation of apoptosis, while the suppression of reactive oxygen species (ROS) inhibits the proliferation of cells to prevent DNA damage. Furthermore, Amalia et al. reported that a BP sample from Indonesia (*Trigona* spp) had an antiproliferative effect on human cancer cell lines with an IC_50_ (half-maximal inhibitory concentration) value (18.6 *±* 0.03 mg/mL) in a time and dose-dependent manner after 24 h. Additionally, BP possesses very low toxicity rather than water-soluble propolis toward normal cells (120, 172). Ravishankar et al. studied that the BP component called quercetin may have anticancer activity by upregulating tumor suppressor genes while disrupting oncogene expression. BP samples (*Olea europaea* and *Coriandrum* spp.) from Morocco have been used as an anticancer agent against HeLa (cervical carcinoma) and MCF-7 (breast adenocarcinoma) cell lines, respectively, and their anticancer activity may be associated with the presence of flavonoids, i.e., kaempferol-3-*O*-rhamnoside and quercetin-*O*- di-glucoside (173). Studies revealed that the BP sample (collected from South Korea) was used against various human cancer cell lines such as NCI-H727 (human lung carcinoma), PC-3 (human prostate adenocarcinoma), AGS (human gastric adenocarcinoma), MCF-7 (breast adenocarcinoma), and A549 (human lung carcinoma) which showed IC_50_ value ranging from 0.9 to >25 mg/mL (174, 175).

The BP protein called hydrolysate (enzymatically cleaved BP protein) can have strong anticancer activity by inhibiting human bronchogenic carcinoma (ChaGo-K1 cells), resulting in IC_50_ value of 1.37 μg/mL (174, 176). In another study, BP along with doxorubicin (DOX) was tested synergistically on mice models having induced breast tumor (4 T1 tumor cells) for 35 days. The blood was taken on 36th day in order to quantify the level of nitric oxide, cytokines, progesterone, estrogen, and testosterone. In addition, it was noted that both BP and DOX work synergistically and upregulate the expression of apoptotic genes and proteins and eventually decrease the volume of tumor cells while decreasing the level of nitric oxide and estrogen and downregulating the level of pro-inflammatory cytokines in mice models with induced 4 T1 breast tumors. BP isoflavones have been used as anticancer agent against breast cancer cells such as triple-negative breast cancer (TNBC) and restrain the activity of endogenous estrogen (177). Research has shown that isoflavones and flavonoids of BP restrain the cell cycle at G1 and or G2/M stages and upregulate the expression of pro-apoptotic genes and dysfunction of the capability of enzymes associated with the growth and development of tumor cells while hindering the activity of intermediates such as fibroblast growth factor (FGF) and vascular endothelial growth factor (VEGF) and also inhibiting the production of ROS (177, 178). Wang et al. concluded that BP polysaccharides (extracted from *Rosa rugosa*) exhibited anticancer activity *in vitro* against HCT116 (human colon carcinoma) and HT-29 (colon cancer) cells in a dose-dependent way (179, 180). Furthermore, BP samples of three stingless bees, i.e., *Heterotrigona itama, Tetrigona apicalis,* and *Geniotrigona thoracica* (from Malaysia) were tested *in vitro* upon MCF-7 (breast adenocarcinoma human cell lines) and MCF-10A (mammary epithelial human cell lines), which inhibits the proliferation of both tumor cell lines in a dose-dependent way (181).

## 5 Bee pollen and honey bee health

There are 20 biogenic amino acids, but honey bee requires only 10 amino acids that are essential to their diet. Different plant origins of pollen grains contain all essential amino acids in fewer amount (89, 182). Except for nutrition, honey bees use those amino acids for maintaining individual immunity and social behavioral patterns. The queen royal jelly as a protein-rich diet can enhance ovarian development and egg production in honey bees (*A. mellifera*) and worker bees since pollen is their main protein source, and its digestibility and quality might be important nutritive factors that determine reproductive capability (183). The most important elements of honey bee nutrition are carbohydrates, proteins, amino acids, and lipids, all of which have a significant impact on individual and colony health (184, 185). The nutrition value of these (carbohydrates, proteins, amino acids, and lipids) contents of some pollen types varies between species (186, 187). In *A. mellifera scutellata* workers, the effect of two types of pollen was examined: aloe (*Aloe greatheadii* var. *davyana*) and sunflower (*Helianthus annuus*) were compared in the field conditions with the queen, and when feeding on aloe pollen, the workers have exhibited greater ovarian development (183). Maize pollen is thought to be lacking essential amino acids and proteins and is believed to be a minor bee feed source (188).

A healthy honey bee colony can consume up to 7 kg of pollen per year. During times of scarcity, it is crucial for a bee colony to have supply of pollen as it greatly impacts their survival and reproductive abilities, such as activating ovarian function of the queen bee. Fermented bee pollen also has the added benefit of inhibiting the growth of harmful bacteria such as *Melissococcus plutonius* (European foulbrood) and *Paenibacillus larvae* (American foulbrood) due to the presence of dodecanoic acid, linoleic acid, myristic acid, and linolenic acids (110). There is a correlation between the reproduction of honey bee colonies and the protein content found in pollen. Honey bees that collect pollen with high protein content tend to have higher colony reproduction rates and bee brood production. Pollen collected from plants that bloom during the spring season contains a higher protein content (average of 24.2%) compared with those that bloom during the summer and autumn seasons (average of 19.3 and 20.5%, respectively). During the spring season, pollen containing protein content of more than 27% enables honey bee colonies to enhance their reproduction (189). Beekeepers need to provide pollen patties as a supplement to bee colonies during pollen deficient season because pollen is the main protein source for honey bees. Additionally, it is also recommended that beekeeper should move their colonies to pollen-rich areas because brood production and development and also the reproductive capability of bees are solely dependent on pollen availability of different plants.

## 6 Bee pollen availability and its consumption

Nowadays, it is a developing business for the beekeeping industry, as BP is regarded as a functional food for human health. To collect pollen from bees, various types of pollen-collecting traps are designed (with bee size small holes) and transplanted with the hive entrance gates for the purpose of detaching pollen from the legs of bees when they enter the hive (190, 191). It is consumed after drying to guarantee long-term safety and stability and is mostly commercialized. If pollen is dried upon heat (more than 40°C to 50°C), it affects the phenolic contents and pollen organoleptic features (191, 192). An alternative way to preserve its nutritional content and organoleptic properties might be freezing (191). BP is a nutrient-rich supplement and has a beneficial impact on health (193). It was reported that the consumption of pollen by women can be considered a source of good health, a strong body, and beauty in old history. Routine use of BP prevents the body from harmful radiotherapy and chemotherapy reactions. It is also useful for people doing hard physical work and children with appetite loss and patients who are starving. Various pollen products can be found in the form of oral liquids, candy bars, tonics, pellets, tablets capsules, powders, and granules in the market (25).

BP can be mixed with biscuits, which increases its protein, sugar, phenolics, and fiber content and also enhances the antioxidant efficacy of biscuits. As a supplemental food, BP added wheat flour in bakeries to produce various food items (4, 14). The recommended dose of pollen consumption for an adult human should range from 20 to 40 g daily (3, 11, 20, 24, 194). Athlete players use BP as a highly nutritive food to increase their athletic ability (47). However, dried or fresh pollen grains frequently have a hard shell that can affect significantly the digestive enzyme penetration into pellets of pollen (194). Bee bread (fermented BP) is said to be more nutritive as compared with BP because the content of some of their valuable components increases, which are highly digestible and penetrable through the gastrointestinal tract (4). Furthermore, the consumption of BP also has significant effect on the beneficial microbiota of animal gut which further helps in food digestion and assimilation and strengthens immunity (46). Thus, it is recommended to use fermented pollen which is more acceptable for nutrient absorption by the digestive tract of humans (194, 195) because microbes degrade the external pollen walls during fermentation, and its internal nutrients can easily be consumed (8).

## 7 Bee pollen and human health risk

In additiob to its beneficial nutrients, some harmful substances such as allergens and other toxic (mycotoxin) compounds are also found in BP. The allergic substances present in pollen are highly hazardous for sensitive people if they consume or inhale pollen. Special care must be taken during pollen consumption and can be utilized according to the physician’s suggestions. BP is an important apicultural product, which is used as a nutritive ingredient and mixed with other products for therapeutic purposes. Alongside its beneficial properties, BP also possesses environmentally hazardous contaminants such as pesticides, toxic elements, and other health risk factors, which can be dangerous both for bee’s life and indirectly for public health (82, 85, 196). During the collection of pollen, environmental contaminants are also attached to the forager body and are transferred to their hives, so beehive products can be used as an environmental contaminants’ indicator (196–199). A total of 189 samples of BP were examined for pesticide adulteration in China, which showed the detection of insecticides such as chlorpyrifos, fenpropathrin, thiamethoxam, imidacloprid, and bifenthrin and fungicides such as triadimefon and carbendazim while acaricides such as fluvalinate and coumaphos were also found in BP samples (85). In a recent study, the researcher examined 45 different samples of BP from various regions of Greece, which exhibited the presence of harmful elements such as arsenic, cadmium, mercury, and lead while some pesticides such as coumaphos (22%) cypermethrin, propargite, dimethoate, and azoxystrobin were also detected (82). The researcher collected BP samples from 13 sites in northern Italy (during the flowering season 2019 to 2020) and examined for various pesticide compounds (insecticides, fungicides, herbicides, and acaricides). They found 97 different pesticides, i.e., mostly fungicides but highly toxic acaricides and insecticides (organophosphates and neonicotinoids), but the concentration of herbicides was low in the tested BP samples. The fungicide called zoxamide affects the motor and cytoskeleton proteins while penconazon and spiroxamine (fungicides) also have a negative impact on the synthesis of sterol in membranes. Another fungicide of most concern is called carbendazim which has been officially prohibited in EU countries since 2014 but is still found in BP samples from Italy (200).

It was reported that the acaricides (mostly used by beekeepers for the controlling of varroa mites) called tau-fluvalinate and coumaphos were found in BP samples of 5.22–85.22 μg/kg and 5.13–39.81 μg/kg, respectively (201). Additionally, 145 BP samples were collected from 10 honey bee colonies in Brazil (São Paulo State) and were analyzed for pesticide residues. The experts identified two pesticides, namely, pendimethalin and bioallethrin in the tested BP samples (199). Furthermore, the consumption of BP also creates some clinical problems such as allergic reactions in pollen-allergic sensitive individuals. However, the allergenicity can be overcome due to the treatment of BP with specific enzymes such as pectinase, papain, and cellulase. Additionally, enzyme-treated BP can mitigate the allergenicity of BP and harmonize the composition of gut microbe (23). BP also contains some fungal species such as *Cladosporium, Alternaria*, and *Aspergillus*, which cause allergic responses (85, 162). Other mycotoxin-producing fungal species were also detected in BP samples such as *Fusarium* spp. and *Penicillium* spp. Studies revealed that there are different types of mycotoxin which are found in pollen samples such as AFB1 (aflatoxin B1), ZEN (zearalenone), OTA (ochratoxin A), T-2, and DON (deoxynivalenol) (202), but the most important and harmful mycotoxin is AFB1 both for animal and human health causing DNA and chromosomal damage (202, 203).

Similarly, mycotoxin T2 and DON have detrimental effects on neurotransmitter and metabolic activities while ZEN and OTA mycotoxins possess neurotoxicity, hepatotoxicity, and immunotoxin effects. In addition, multifloral BP showed a higher concentration of mycotoxin than monofloral BP, whereas bee bread exhibited maximum amount of mycotoxins in analyzed samples (202). Another study from Lithuania stated that different fungi genera such as *Yeast*, *Cladosporium, Alternaria*, and *Fusarium* spp. were found in 45 samples of BP after 3 days of storage condition along with the presence of mycotoxin DON (120 μg/kg) and ZEN (280 μg/kg) (204). Researchers also detected various molds in BP samples from Ukraine Serbia, and Slovakia, such as *Rhizopus, Aspergillus, Alternaria, Fusarium, Penicillium.* and *Mucor* (196). In the same way, along with fungi, other allergens such as pyrrolizidine alkaloids and toxic elements are also present in BP. Mostly, pollen-allergic people show more clinical symptoms when they inhale airborne pollen, such as hay fever, infection of oral mucosa, skin, and cardiovascular and respiratory tract septicity. But allergic reactions after the consumption of pollen are rare. The allergenicity of pollen depends on the temperature and pH value because the main allergens present in pollen are glycoproteins (water-soluble proteins), which can easily diffuse after contact with airways mucosa (1).

The pollen allergic individuals should follow physician precaution measures while using other bee products (205). The presence of pyrrolizidine alkaloids in BP can create severe conditions of lung cancer and hepatotoxicity, which are strongly related to the botanical origin of BP. Additionally, pyrrolizidine alkaloid-producing plants belong to three botanical families, namely, Fabaceae, Boraginaceae, and Asteraceae. The BP of the plant species called *Echium vulgare* possesses high pyrrolizidine alkaloid contents in most European countries (1, 206), while the toxic elements include aluminum, lead, strontium, cadmium, arsenic, chromium, nickel, and mercury, which can cause neurotoxicity and other abnormalities (1, 207). The occurrence and bioaccumulation of heavy metals in the human physique create rigorous problems, such as intellectual disability, carcinogenesis, and other body abnormalities, such as disruption of metabolism and growth and nervous disabilities (85). The use of BP in food products and pharmaceutical and cosmetics industries considered as a vital bee product worldwide. Further research and clinical trials of BP *in vitro* and *in vivo* are needed to overcome the adverse effects of BP and eliminate its undesirable consequences on human health.

## 8 Honey bee products and nanotechnology/nanoparticles

Honey bee products have been used as a natural medicine and food supplement since the ancient era. These valuable products were used not only by humans but also utilized for the benefit of other animals. The way of its use is different, depending on the type of bee product and treatment procedure or consumption, such as injection, tablets, syrup, balm, or in conjugation with other products. Like other medicine or pharmaceutical products, api-products also have some side effects or are dangerous for allergic individuals. To overcome these difficulties, scientists are trying to use bee products in nanotechnology, which are safe, effective, and target-specific. Nanotechnology or nanoparticles (NPs) is a modern technique, where a specific carrier or vector (containing medicine) is used to target a specific site within the body of a patient. Different bee products such as bee venom, BP, propolis, royal jelly, and honey were incorporated into nanoparticles, which showed effective results and safe deliveries in many trial studies. Soman et al. used melittin (bee venom component) in perfluorocarbon nanoparticles against tumor cells and exhibited successful results (208).

Additionally, the main advantage of nanoparticles is that it protects the holding product from degrading enzymes in bloodstream, enhances bio-dispersal, and is target specific. Huang et al. administered alpha-melittin nanoparticles (α-MEL-NPs) and injected intravenously with a concentration of 20 mg/kg which showed the growth inhibition of B16F10 (human melanoma cells) and have no toxic effects on normal cells (209). Furthermore, the hemolytic activity of melittin in nanoparticles reduces by approximately 90% to direct utilization, enhances circulation duration, and gradually releases in a target site (210). The fungal chitosan (an amino polysaccharide derived from *Fusarium oxysporum*) loaded with bee venom nanoparticles was an effective anti-tumor agent for cervix carcinoma (HeLa cells) (211). In addition, chitosan nanoparticles containing BV possess inhibitory activity toward many clinical fungal strains, such as *Kodamaea ohmeri*, *Cryptococcus neoformans*, and *Candida albicans* (212). In another study, Saber et al. successfully administered chitosan nanoparticles, holding bee venom for the treatment of diseases caused by amoeba in the mice model (174).

Similarly, BP nanoparticles showed anti-cancer activity against A549 cancer cells (lung cancer cell lines) (213). It was documented that BP silver-based nanoparticles (AgNPs) have anti-diabetic properties and inhibit α-glucosidase and α-amylase enzymes more effectively than treatment with BP extract (214). Additionally, AgNPs prepared with honey bee extract (12–18 nm size) were used against colon cancer cells, which revealed anti-cancer activity (215). AgNPs containing an aqueous solution of rapeseed pollen exhibited anti-cancer capability against MCF-7 and MDA-MB-231 cancer cell lines (216). It has been studied that the magnetite nanoparticles (Fe_3_O_4_/PABA/MNPs) coated with BP extract could have anti-microbial activity toward gram-positive bacteria and gram-negative bacteria and some fungal species (217). Moreover, ethanolic extract of propolis-containing polymeric nanoparticles has anti-fungal activity against *Candida albicans* (174). Do Nascimento et al. concluded that ethanolic extract of Brazilian red propolis loaded with polymeric nanoparticles possesses anti-leishmanicidal capability (174, 218). Studies revealed that selenium nanoparticles (SeNPs) conjugated with ethanolic extract of propolis (Indian origin) possess antioxidant and anti-biotic and anti-fungal properties (219).

Furthermore, AgNPs loaded with propolis of stingless bees showed anti-cancer action against A549 cell lines (human lung cancer cells) with an inhibitory concentration (IC50) value of 38 μg/mL (216). It has been stated that zinc oxide (ZnO) nanoparticles in conjugation with bacterial cellulose and propolis extract revealed combined anti-microbial activity toward *Candida albicans, Bacillus subtilis,* and *Escherichia coli* (220). In another study, royal jelly-loaded AgNPs showed antibacterial activity against *S. aureus* (gram-positive bacteria) and *S. typhimurium* (gram-negative bacteria). The author further stated that the inhibitory effect of royal jelly-based AgNPs was more on *S. typhimurium* than on *S. aureus* (221). Similarly, AgNPs containing royal jelly also showed stronger antibiotic activity against *E. coli* than *B. subtilis* (222). Research has shown that nano-silver with royal jelly can have anti-inflammatory action, which enhances the activity of immune cells in mice models (223).

## 9 Conclusion and future remarks

BP is a nutritional product and an excellent apitherapeutic agent for different health problems. It contains many beneficial components which are necessary for health maintenance and can be used in different recopies because of its antioxidant capacity for long-lasting preservation of fats. BP is also used as an immunity booster, and its beneficial effects on reproduction. Its quality varies with botanical origin and geographic area conditions. BP shows significant therapeutic effects when experimented on different organisms against different health issues. It is a natural food and medicine, and it needs much attention as a health-oriented product. Future recommendations suggest its use in different types of apitherapy for the treatment of different human and animal diseases and physiological alterations. Few countries have regulation strategies for the usage of honey bee products such as China, Argentina, Brazil, Switzerland, and Poland. There must be legislative principles to standardize honey bee products as supplemental food and minimize the contaminants for safe and healthy use. It is important to prioritize the health and safety of honey bees by protecting their foraging areas and habitats and minimizing human impact.

## Author contributions

SA: Conceptualization, Supervision, Visualization, Writing – original draft, Writing – review & editing. AU: Conceptualization, Data curation, Validation, Writing – original draft, Writing – review & editing. FG: Conceptualization, Methodology, Project administration, Writing – original draft. GR: Software, Supervision, Visualization, Writing – review & editing. MK: Conceptualization, Data curation, Formal analysis, Writing – review & editing. MH: Conceptualization, Methodology, Project administration, Writing – review & editing. AA: Writing – review & editing, Supervision, Validation, Funding acquisition. C-CC: Writing – review & editing, Supervision, Funding acquisition. IT: Conceptualization, Investigation, Project administration, Supervision, Visualization, Writing – review & editing.
